# Aging and temporal integration in the visual perception of object shape

**DOI:** 10.1038/s41598-023-40068-x

**Published:** 2023-08-07

**Authors:** J. Farley Norman, Jessica L. Lewis, Emily N. Bryant, Juma D. Conn

**Affiliations:** 1 Department of Psychological Sciences, Ogden College of Science and Engineering, Western Kentucky University, 1906 College Heights Blvd. #22030, Bowling Green, KY 42101-2030 USA; 2 Center for Applied Science in Health and Aging, Western Kentucky University, Bowling Green, KY 42101-2030 USA; 3 Carol Martin Gatton Academy of Mathematics and Science, Bowling Green, KY USA

**Keywords:** Human behaviour, Perception

## Abstract

It has been known for more than 160 years that highly occluded objects that would normally be visually unrecognizable can be successfully identified when they move. This *anorthoscopic* perception relies on the visual system’s ability to integrate information over time to complete the perception of an entire object’s shape. In this experiment, 16 younger and older adults (mean ages were 20.5 and 74.6 years, respectively) were familiarized with the (unoccluded) shapes of five naturally-shaped objects (bell peppers, *Capsicum annuum*) until they could be easily identified (i.e., with accuracies of at least 90 percent correct). All observers then viewed the stimulus objects anorthoscopically as they moved behind narrow slits; only small object fragments could be seen at any given time, because the objects were almost totally occluded from view. Even though the object identification performance for all observers was equivalent when whole object shapes were visible, a large age-related deficit in object identification emerged during anorthoscopic viewing such that the younger adults’ identification performance was 45.4 percent higher than that of the older adults. This first ever study of aging and anorthoscopic perception demonstrates that there is an age-related deficit in performing the temporal integration needed for successful object recognition.

## Introduction

**Figure 1:**
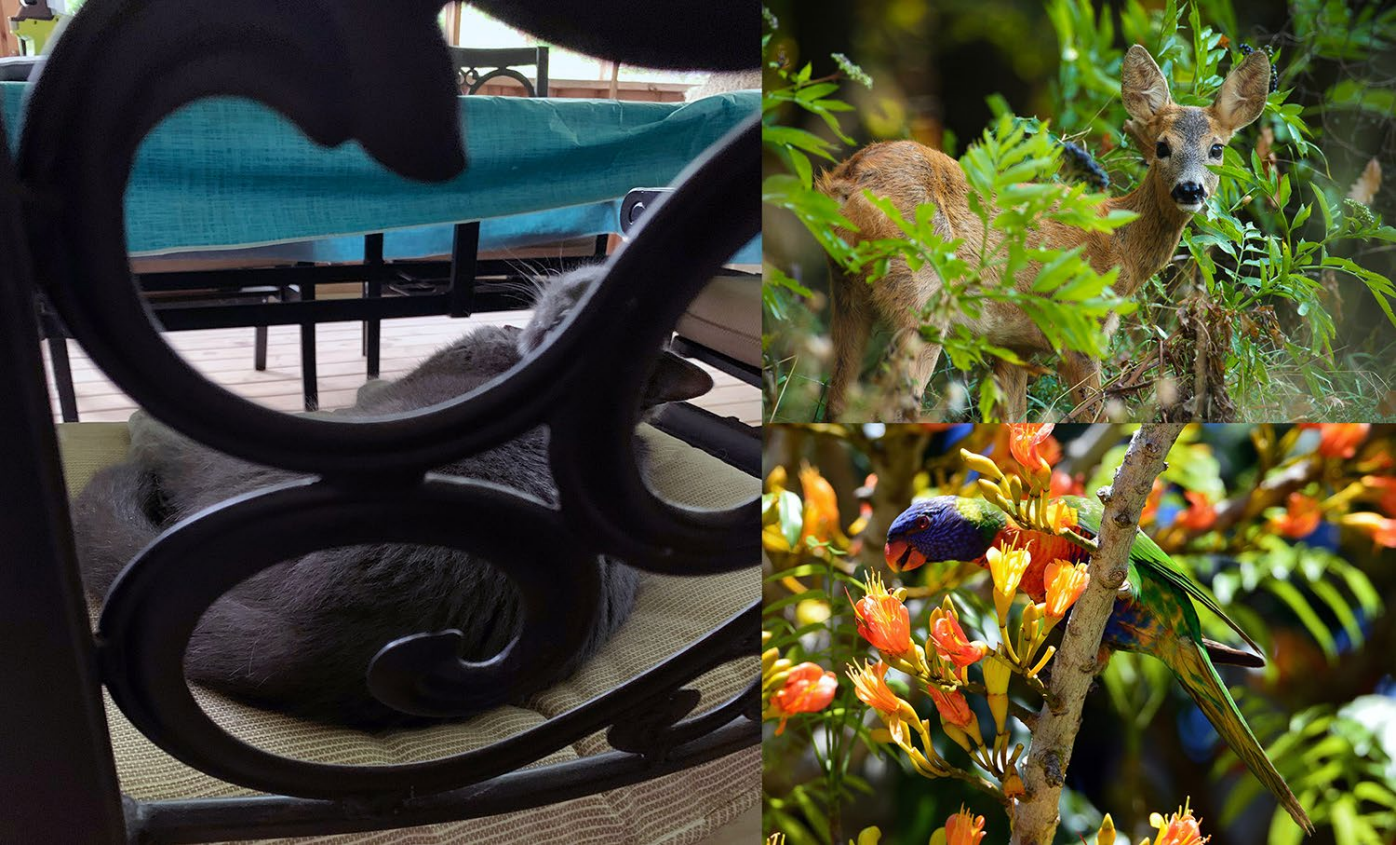
Photographs of three objects (cat, deer, & parrot) that have been partially occluded due to intervening portions of a metal chair, tree branches, leaves, and flowers. Such occlusions happen frequently in natural and everyday environments. The photograph of the cat (left) was taken by the first author (J.F.N.) using an iPhone Xr digital camera, while the photographs of the deer (upper right) and parrot (lower right) have been licensed from Adobe Stock Images (images 188,230,887 & 469,052,338).

**Figure 2:**
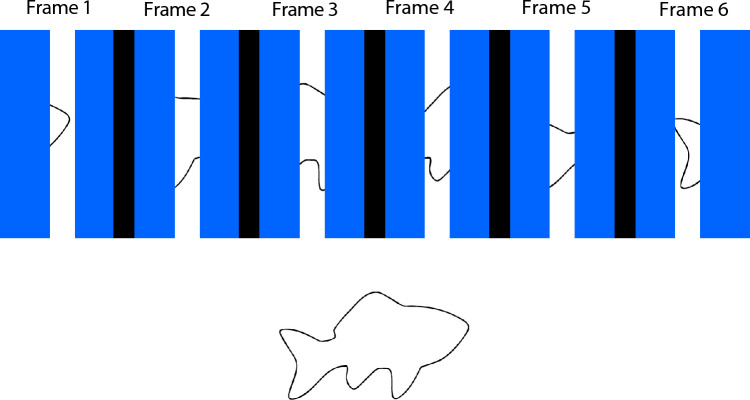
Six frames of an apparent motion sequence as a partially occluded object (a fish, see bottom) moves (from left to right) behind a blue occluding surface. Because only small pieces of the moving object can be seen through the narrow slit/aperture in the occluding surface, it is difficult to recognize.

One of the most important aspects of human and mammalian vision is our ability to recognize and identify objects from incomplete information. In both natural and manmade environments, our view of objects is often partially blocked by occluding surfaces. Consider, for example, Fig. 1. We can easily recognize the cat, deer, and parrot despite the fact that significant proportions of all three animals are not visible, because of intervening surfaces, leaves, branches, and flowers. In cases when objects move behind occluding surfaces, human observers can successfully recognize objects even when they are more than ninety percent occluded^1, 2^. This ability was first studied more than 160 years ago by Zöllner^3^; he described a phenomenon that he referred to as “anorthoscopic” perception. In his demonstration he moved simple figures, such as squares or circles, behind a narrow vertically-oriented slit (2 mm wide × 40 mm tall). Because the narrow width of the slit was much smaller than the overall size of the figures, only a small portion of each object was visible at any given moment in time. Nevertheless, Zöllner found that when these figures were moved rapidly back and forth behind the slit, the whole shape of the squares and circles could be perceived (despite some visible compression or expansion of the shape along the direction of movement). Zöllner’s observation was important, because it demonstrated that the human visual system is capable of integrating (over time) the momentarily visible fragments into a complete perception of whole objects. Later investigations have confirmed Zöllner’s observations^1, 2, 4–7^. As an example, consider Fig. 2. At the top, an outline object (defined by black contours) translates from left to right behind a narrow slit (the surface occluding most of the object is blue). The top panel indicates six frames of an apparent motion sequence. It is important to note that in any particular frame (i.e., moment of time), only a meaningless fragment is seen, and participants would ordinarily be unable to identify the object. However, if participants are allowed to see an entire motion sequence of such views^1–7^, they would easily be able to perceive the whole object as a fish (shown at the bottom of Fig. 2). Another good example of effective temporal integration was provided about 100 years after Zöllner by Eriksen and Collins^8^. On any given trial in their experiment two seemingly random sets of dots were presented sequentially with interstimulus intervals (ISI’s) that varied between 25 and 100 ms. No meaningful pattern could be seen in either the first or second dot pattern. Nevertheless, when the two dot patterns were shown in succession, observers could see three-letter nonsense syllables (such as HOV). There were 20 possible nonsense syllable stimuli; therefore, chance identification performance was five percent correct. The observers’ identification performance was very high, approximately 85 percent correct for ISI’s of 25 ms. This performance decreased to about 42 percent correct (still much higher than chance) for ISI’s of 100 ms.

In nearly all of the published research on anorthoscopic perception to date^1, 3–7^, the depicted figures have been 2-dimensional (2-D) objects, drawings, or contours, and they were simply translated back and forth behind the slit (with the slit and depicted figures being presented in a fronto-parallel plane). In 2021, Norman et al.^2^ found that observers can identify and discriminate the 3-dimensional (3-D) shapes of solid objects that rotate in depth behind narrow slits in an occluding surface, thus demonstrating that the human visual system can temporally integrate meaningless fragments of visible surfaces into coherent perceptions of 3-D object shape. The phenomenon discovered by Zöllner^3^ thus generalizes from 2-D figures to the perception of solid (i.e., 3-D) object shape.

In a prior study involving aging and motion, Andersen and Ni^9^ required younger and older observers to judge the shape of moving objects, where the object shape was defined by the accretion and deletion of texture (e.g., Gibson^10^, pp. 203–205) as the textured stimuli translated across a similarly textured background. When performance was compared across experimental conditions where the rate of occlusion events was constant, the performance of the older adults was more negatively affected by reductions in texture element density. The older adults’ shape recognition performance was not differentially affected, however, when the stimulus speed was varied (which produced variations in the rate of texture accretion and deletion). From these results, Andersen and Ni^9^ concluded (p. 116) by saying “the results of the present study indicate age related decrements in spatial but not temporal integration in identifying 2D shape from kinetic occlusion”.

The finding by Andersen and Ni^9^ that aging does not affect temporal integration (at least in the context of shape recognition from kinetic occlusion) suggests that older adults might perform well when anorthoscopically perceiving object shape. It is also true, however, that aging is associated with significant reductions in the ability to perceive basic aspects of motion, such as direction and speed^11–19^. The purpose of the current experiment is straightforward, to evaluate whether older adults can perform the temporal integration needed to recognize meaningful solid objects from the momentarily visible fragments that occur when moving objects are viewed through apertures in occluding surfaces. The current experiment is the first study to ever evaluate whether and to what extent older adults can perceive anorthoscopic shape.

## Method

### Apparatus

An Apple Mac Pro computer (Dual Quad-Core processors, with ATI Radeon HD 5770 hardware-accelerated graphics) was used to present the apparent motion sequences and to record the observers’ responses. The stimulus displays were presented on an Apple 27-inch LED Cinema Display, and were viewed from a distance of 90 cm.

### Experimental stimuli

**Figure 3:**
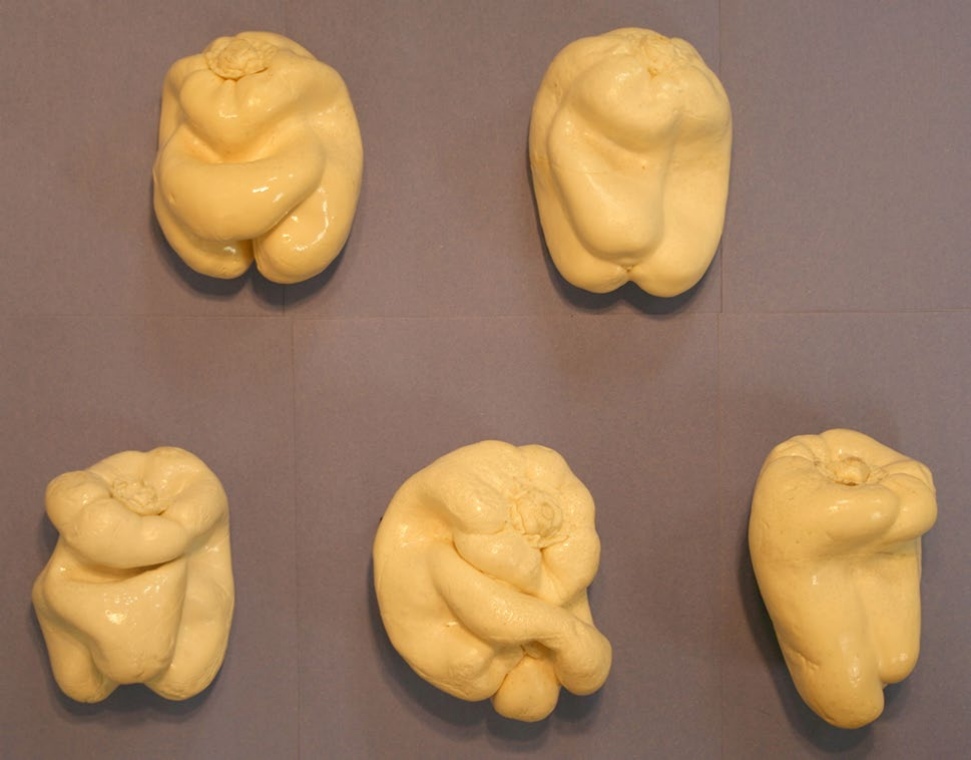
A photograph of the five natural solid objects (bell peppers, *Capsicum annuum*) used as stimuli in the current experiment. Objects 1 through 5 are located from the upper left to bottom right. This photograph was taken by the first author (J.F.N.) using a Canon Rebel XTi digital SLR (single-lens reflex) camera.

**Figure 4:**
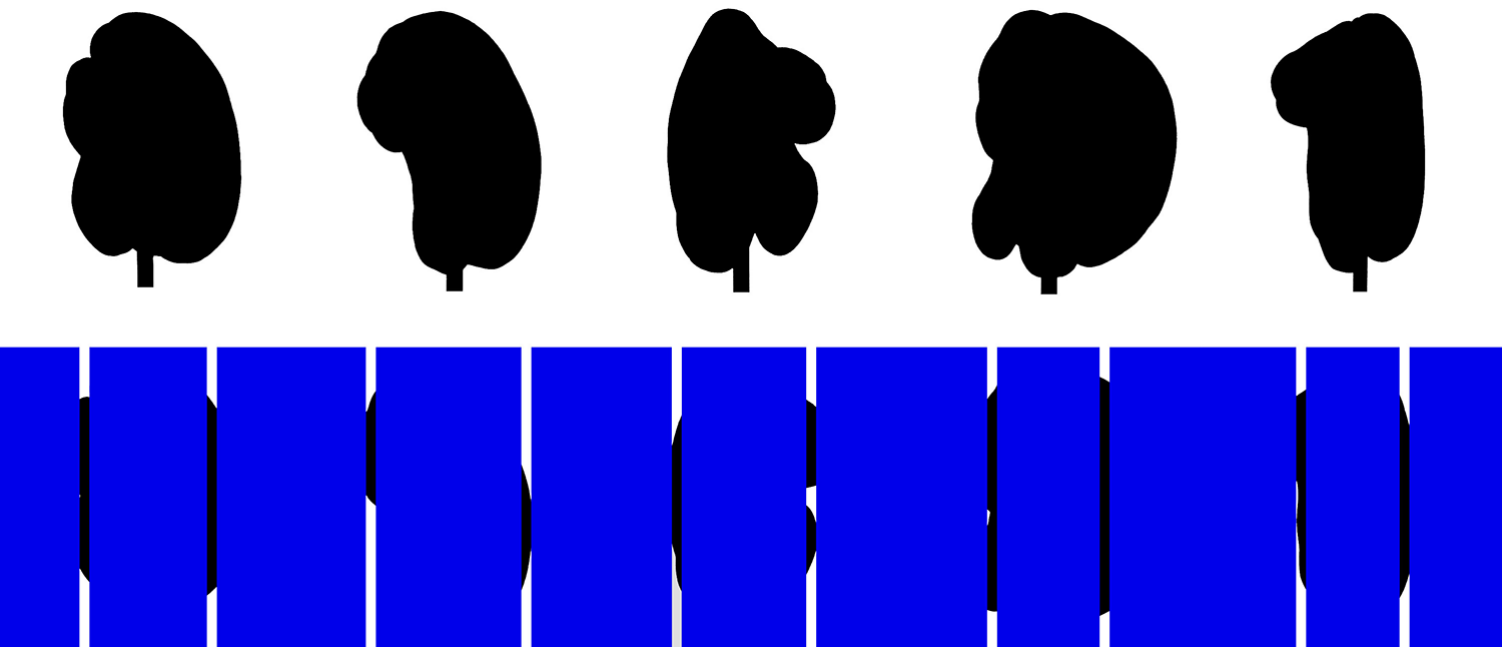
Example static views of the experimental stimuli. The top row shows representative silhouettes of objects 1–5 (left to right), while the bottom row shows those same object silhouettes as they would be seen through narrow vertically-oriented slits/apertures in a (blue) occluding surface. One can readily see that the occluding surface blocks more than ninety percent of the object shapes from view, so that only small pieces of the stimulus objects can be seen at any given time.

The stimulus displays were identical to the moving stimuli used previously by Norman et al.^2^. The 3-D shapes of the solid objects (plastic replicas of 5 naturally-shaped bell peppers, *Capsicum annuum*, see Fig. 3 and Norman et al.^20^) were defined by the *kinetic depth effect*^21–23^—i.e., by the deformations in the projected silhouettes/cast shadows of the solid objects that occurred as they rotated in depth about a vertical Cartesian axis. The objects rotated 4.5° at each frame transition, such that a complete revolution occurred every 80 frames. The frames of the apparent motion sequences were updated at 60 Hz, and a total of 180 frames were presented on each trial. The duration of each stimulus presentation was, therefore, 3.0 s. It is important to keep in mind that the viewing of the solid object stimuli was always *anorthoscopic*^1–7^ in the experiment; only bits and pieces of the object silhouettes were momentarily visible through rectangular apertures (slits) as the stimulus objects rotated in depth behind an occluding surface. This is illustrated in Fig. 4. In the current experiment, two slits (4 mm wide) were used, one on each side (left and right) of the axis of object rotation. The horizontal position of the slits was randomly varied on each trial (the offset from the axis of object rotation ranged from 20 to 32 mm) so that even when the same stimulus object was presented on multiple occasions, the exact proximal stimulus at the observers’ eye was always different.

### Procedure

Each observer participated in three experimental sessions of 100 trials (20 trials for each of the 5 stimulus objects, all presented in a completely random order). Each session took about 10–12 min. After the three experimental sessions, each observer had, therefore, made a total of 300 judgments (60 trials for each of the stimulus objects). All observers were allowed and encouraged to take a break in between sessions. After each trial, the observers’ task was to simply identify the object (i.e., whether it was object 1, object 2, object 3, etc.). During these experimental sessions, no feedback regarding their accuracy was ever provided to the observers.

While the task itself was straightforward, it is very important to keep in mind that before the experiment, the observers were completely unfamiliar with the unique shapes of these five bell pepper objects (Fig. 3). Without adequate familiarization, it would be impossible for observers to perform the task at all (no one could respond “object 1”, for example, on any given trial if they did not know what object one looks like). Therefore, to enable the task, all observers were familiarized (as was done in three previously published studies^2, 22, 24^) with these five objects. First, the observers were allowed to look at the actual physical objects themselves (Fig. 3); if they wished, they were allowed to pick the objects up and examine them. After this, the observers were presented with apparent motion sequences depicting rotations of the whole object silhouettes (i.e., no slits/anorthoscopic viewing, similar to the stimuli presented in the top row of Fig. 4) until they could easily recognize the stimulus objects (i.e., with at least 90 percent correct accuracy). At the beginning of each experimental session, the observers were repeatedly presented with blocks of 10 trials (all 5 stimulus objects presented twice in a random order, *with* feedback) until their identification performance was 90 percent correct or better. This procedure ensured that every single observer, younger and older, was equally good at recognizing the stimulus objects prior to anorthoscopic viewing for each experimental session. No one ever saw the stimulus objects presented behind slits in occluding surfaces until they had effectively learned which object was object 1, which was object 2, etc.

### Observers

If an age-related deficit does exist with regards to the anorthoscopic perception of solid object shape and if it is as large as the age effect found in a recent experiment of ours (see Experiment 1 of Norman et al.^25^) involving the perception of 3-D shape from motion, a power analysis reveals that we would need a total of 16 observers (8 younger adults and 8 older adults) to have a 90 percent chance of detecting it. We therefore recruited 16 naive observers to participate in the experiment (8 younger and 8 older). The mean ages of the younger and older observers were 20.5 years (ages ranged from 18 to 25 years, sd = 2.4) and 74.6 years (ages ranged from 68 to 77 years, sd = 3.0), respectively. The observers’ visual acuity was excellent; the acuity of the younger and older observers measured at 100 cm was − 0.11 and − 0.08 LogMAR (log minimum angle of resolution), respectively (zero LogMAR represents normal visual acuity, while negative and positive values represent better than and worse than normal acuity, respectively). The study was approved by the Institutional Review Board of Western Kentucky University, and each participant signed an informed consent document prior to testing. Our research was carried out in accordance with the Code of Ethics of the World Medical Association (Declaration of Helsinki).

## Results

**Figure 5:**
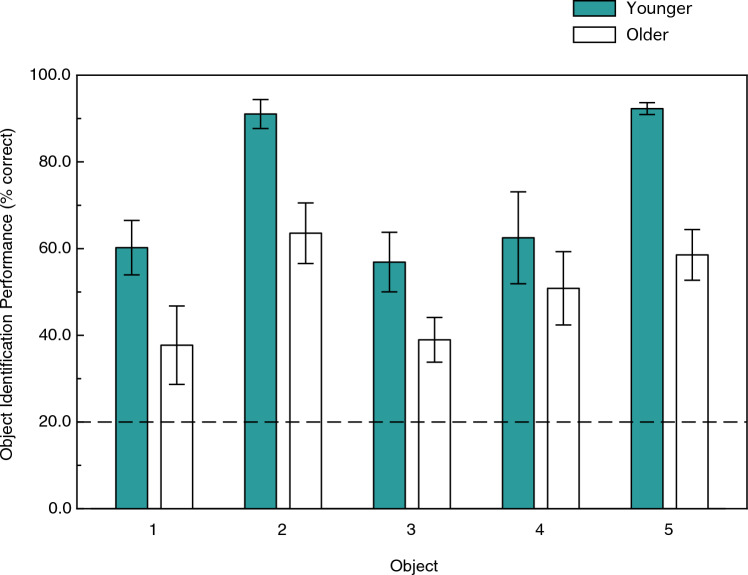
The younger and older observers’ overall object identification accuracies (percent correct values) plotted for each of the 5 stimulus objects. The dashed line indicates chance levels of performance. The error bars indicate ± 1 SE.

The younger and older observers’ object identification performances are shown in Fig. 5. One can readily see that the observers’ identification performance for all objects was much higher than chance, although there were significant variations in performance across the various stimulus objects (F(4, 56) = 13.4, *p* < 0.000001; η^2^_p_ = 0.49), as indicated by a 2 (age) × 5 (object) × 3 (sessions) split-plot analysis of variance (ANOVA). Identification performance was highest for objects 2 and 5 (77.3 & 75.4 overall percent correct, respectively), and was worst for objects 1 and 3 (49.0 & 47.9 overall percent correct, respectively). One can also see a clear effect of age (F(1, 14) = 11.0, *p* = 0.005; η^2^_p_ = 0.44). The younger adults’ object identification performance was 45.4 percent higher than that of the older adults. At this point it is very important to remember that all participants, both younger and older, were able to identify the stimulus objects with a high degree of accuracy (90 percent or better) when there was no aperture/slit (i.e., when they could see the whole unoccluded objects). This adverse effect of age only occurred during anorthoscopic viewing (e.g., bottom row of Fig. 4). The lack of an age x object interaction (F(4, 56) = 1.2, *p* = 0.32; η^2^_p_ = 0.08) indicates that the effect of age was similar in magnitude for all stimulus objects.

**Figure 6:**
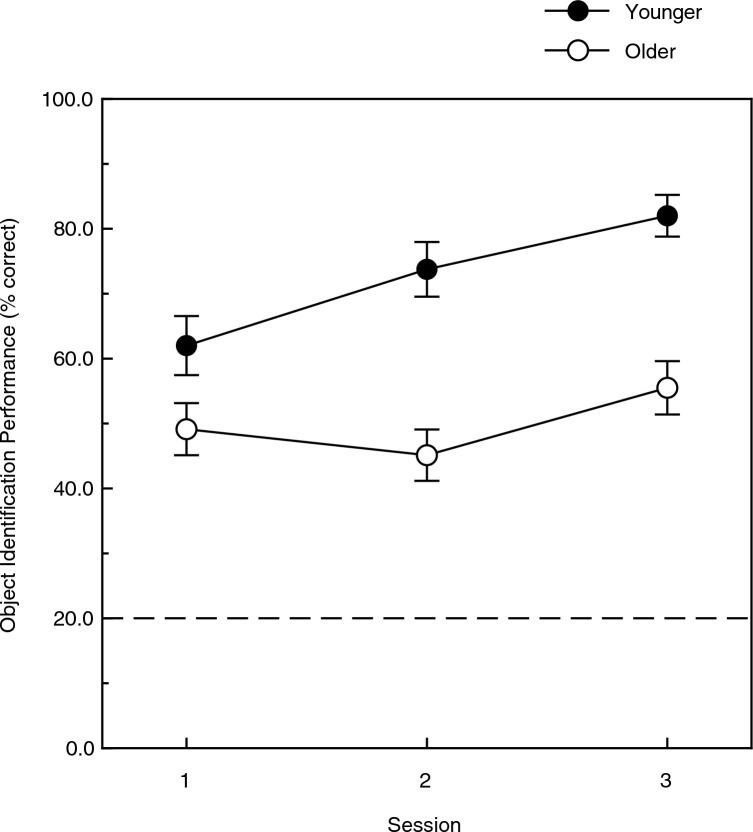
The younger and older observers’ overall object identification accuracies (percent correct values) plotted across the three experimental sessions. The dashed line indicates chance levels of performance. The error bars indicate ± 1 SE.

Figure 6 plots the observers’ object identification performance across the three experimental sessions. The observers’ performance improved across sessions (F(2, 28) = 9.6, *p* < 0.001, η^2^_p_ = 0.41), although the rate of improvement from session 1 to session 3 was different for the younger and older adults (the identification performance was 32.3 and 13.0 percent higher in session 3 than session 1 for the younger and older observers, respectively). This improvement in performance across sessions (especially that exhibited by the younger adults) is similar to that obtained in an earlier study that also used natural objects (bell peppers) as experimental stimuli^26^. Given that the rates of improvement were clearly different for the younger and older adults (see Fig. 6), it is not surprising that the age x sessions interaction was significant (F(2, 28) = 3.8, *p* < 0.04; η^2^_p_ = 0.22).

**Figure 7:**
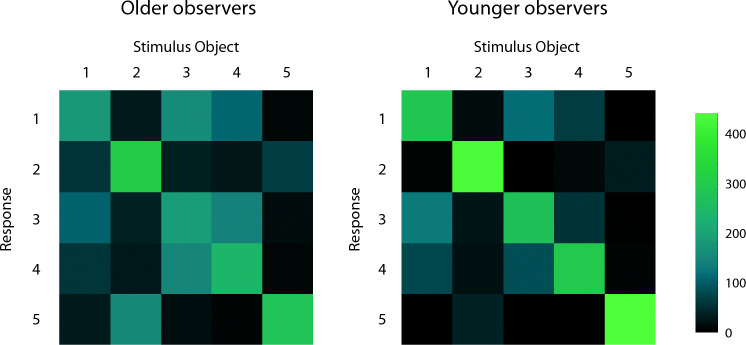
The younger (right panel) and older (left panel) observers’ overall confusion matrices. For each stimulus object, it is possible to readily see to what degree that object was perceptually confused with others. Correct responses are located on the diagonals, while off-diagonal cells indicate errors/confusions. Perfect discrimination performance would be indicated by bright green on the diagonals with black everywhere else.

**Figure 8:**
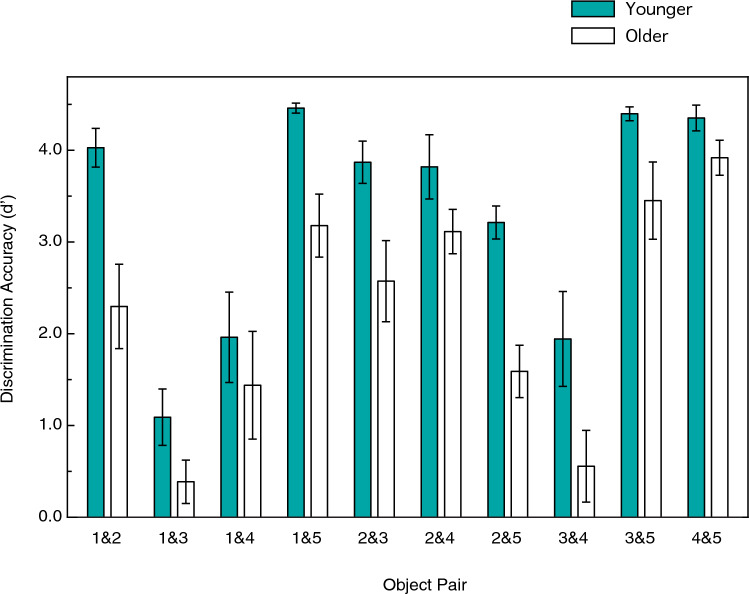
The younger and older observers’ overall object discrimination accuracies (d’ values) plotted for each of the 10 pairs of stimulus objects. Zero indicates chance levels of discrimination ability. The error bars indicate ± 1 SE.

Another important way to evaluate the observers’ performance is to examine pairwise discrimination ability and object confusions. For example, consider Fig. 7. For each stimulus object, it is possible to readily see to what degree that object was perceptually confused with others. Correct responses are located on the diagonals, while off-diagonal cells indicate errors (i.e., object confusions). Perfect discrimination performance would be indicated by bright green on the diagonals with black everywhere else. One can readily see the overall adverse effect of age already mentioned—the green on the diagonal for the younger observers is much brighter and the off-diagonal cells are darker. Therefore, the younger observers produced higher frequencies of correct responses and exhibited less object confusions. One can see both similarities and differences when comparing the performance of the younger and older observers. For example, observers in both age groups confused objects 1 and 3. In many instances, when the observers were anorthoscopically shown object 1, they responded object 3. Likewise, when the observers were anorthoscopically shown object 3, they often responded object 1. The older observers, however, exhibited much more confusion than the younger observers for some of the other object pairs (e.g., objects 2 & 5 and objects 3 & 4). To quantify the observers’ object discrimination performance, we calculated d’ (the perceptual sensitivity measure of signal detection theory^27^) values for each of the 10 pairs of stimulus objects. These object discrimination accuracies are plotted in Fig. 8. The object discriminabilities/confusions vary widely. Some object pairs are easily discriminable (few confusions), such as objects 3 and 5, and objects 4 and 5. On the other hand, other object pairs are very confusable (less discriminable), such as the already mentioned objects 1 and 3. This effect of object pair upon discriminability (F(9, 126) = 43.0, *p* < 0.000001; η^2^_p_ = 0.75) was significant according to a 2 (age) × 10 (object pairs) split-plot ANOVA. The main effect of age was also significant (F(1, 14) = 10.1, *p* = 0.007; η^2^_p_ = 0.42), but the age x object pair interaction was not (F(9, 126) = 1.6, *p* = 0.12; η^2^_p_ = 0.10). This indicates that the magnitude of the effect of age was similar across the 10 object pairs. Overall, the younger observers’ d’ values were 47.2 percent higher than those of the older observers (overall d’ values were 3.313 and 2.250, respectively).

## Discussion

The pioneering study by Andersen and Ni^9^ demonstrated that there was an age-related deficit in spatial, but not temporal, integration, in the context of recognizing 2-D shapes defined by the kinetic accretion and deletion of texture. The intriguing results of this study suggested that older adults might perform well for other shape-related tasks requiring temporal integration. This possibility helped motivate the current experiment. It is true that older adults can anorthoscopically perceive solid object shape (notice, for example, that the older adults’ overall identification performance in Fig. 5 is much higher than chance levels, 49.9 vs. 20.0 percent correct). Our results also demonstrate, however, that aging is accompanied by substantial deficits (see Figs. 5 and 8) in the ability to recognize objects when they move behind occluding surfaces (e.g., Fig. 4).

It is certainly true that good object recognition performance under the present circumstances (anorthoscopic viewing, objects almost completely occluded from view) requires effective integration of information across time (see Fig. 2). Spatial factors are also important, however. Consider, for example, the theory of spatiotemporal relatability developed by Palmer, Kellman, and Shipley^28^. The position updating hypothesis is an important component of this theory; according to this hypothesis (p. 517), “the spatial positions of occluded but persisting object fragments are updated over time as an object moves behind an occluding surface. These occluded, persisting, positionally updated fragments can then be combined with currently visible regions to allow computation of object unity and shape”. If older adults simply possessed reduced temporal integration and/or spatial position updating abilities, this could probably explain an overall difference in performance between younger and older adults (e.g., see Fig. 5), but it could not explain the fact that the older observers in our study exhibited a wider variety of confusions between the various pairs of stimulus objects (see Fig. 7). Object identification/recognition for naturally-shaped solid objects almost certainly requires detecting the unique spatial constellation of surface “features”^29–33^ that are characteristic of a particular object. Almost all surface regions on naturally occurring solid objects are either saddle-shaped (*hyperbolic*), bump- or dimple-like (*elliptic*), or cylindrical (*parabolic*). Many researchers have hypothesized that solid object shape is represented^34–38^ as spatial configurations of these types of surface regions. In our current stimulus displays, only small portions of the objects are visible at any given time because of occluding surfaces (see Fig. 4); if older adults were less able to form adequate representations of the object shapes because of the high amount of occlusion, this could certainly have produced the wider variety of object confusions exhibited by the older observers in the current study.

At this point, it is very important to remember that older adults can perform well when performing many visual tasks—age-related deficits in vision are not universal. For example, under full cue conditions, older adults can visually discriminate solid object shape just as well as younger adults (see results of Experiment 1 of Norman et al.^39^). The judgments of environmental distances by older adults are just as accurate as those made by younger adults and are sometimes even superior^40–43^. In a recent study by Meng et al.^44^, younger and older observers made judgments about visual patterns that required knowledge/perception of Euclidean, affine, projective, and topological geometric properties. While there were age-related differences in reaction time in this study, there were no age-related differences in the accuracy of the observers’ performance. It is clear from this short review that for many visual tasks, older adults perform very well, just as good as younger adults. It is important to remember, however, that substantial age-related deficits frequently occur for tasks involving the judgment of either the speed or direction of motion^11–19^. Furthermore, aging apparently results in a reduction in inhibition within motion-sensitive cortical brain areas^45, 46^; this reduced inhibition produces deficits in such fundamental and important visual abilities as figure-ground segregation^46, 47^. In their 2008 article, Billino et al.^17^ conclude by saying (p. 1260) “our findings support the hypothesis that perceptual capabilities are not equally prone to age-related deterioration”. The current and past results from our laboratory and others continue to support the conclusion of Billino et al.^17^—while the performance exhibited by older adults for some visual tasks (such as the anorthoscopic object recognition task evaluated in the current study) is impaired relative to that of younger adults, increases in age are not accompanied by deficits for other important visual abilities.

## Conclusion

Older adults can anorthoscopically perceive objects, but their visual recognition abilities are nevertheless impaired relative to those of younger adults. Effective behavior in everyday life frequently depends upon vision in general, and visual object recognition in particular. Our findings suggest that older adults may have difficulty in recognizing objects (which thus reduces the likelihood of appropriate responsive behavior) in both cluttered manmade environments and naturalistic environments with dense brush, foliage, etc. —in such common environments, object visibility is impaired due to extensive amounts of occlusion.

## Data Availability

The datasets generated during and/or analyzed during the current study are available from the corresponding author on reasonable request.
